# Supplementary material for: Differences in gene expression may contribute to the racial differences in the risk of MASLD
Source: PLoS One. 2026 May 4;21(5):e0345383. doi: 10.1371/journal.pone.0345383 (PMC13138634; doi:10.1371/journal.pone.0345383)
Supplement: Attachment — Submitted filename: Responses.pdf [file pone.0345383.s005.pdf]

Dear Dr. Shmarakov,

Thank you for the opportunity to revise and resubmit our manuscript entitled “Differences in Gene Expression May Contribute to the Racial Differences in the Risk of MASLD.” We sincerely appreciate the time and effort you and the two anonymous reviewers devoted to providing thoughtful and constructive feedback.

In response to the reviewers’ comments and suggestions, we have substantially revised and expanded the manuscript, with particular emphasis on strengthening and clarifying the Discussion section. We believe that these revisions have significantly improved the clarity, rigor, and overall impact of the work.

Below, we provide a detailed, point-by-point response to each reviewer comment, describing how each concern was addressed in the revised manuscript. We have uploaded two versions of the manuscript: a clean version and a version with highlighted changes for ease of review.

On behalf of all authors,

Ivan Gorlov

Reviewer 1.

While this study addresses important differences to keep into account when analyzing different populations, such as the differences in specific genes driving disease progression, it fails to fully conclude what those genes found signify in that context.

Our goal was less ambitious than “to fully conclude what those genes found signify in that context.” We want to bring attention to the possibility that genetic variation may contribute to ethnic differences in MASLD incidence. The typical explanation of ethnic differences in MASLD refers to the differences in lifestyle. This explanation is likely insufficient, especially when we consider Black population with higher prevalence of lifestyle risk factors and lower MASLD prevalence compared to Whites.

The paper published by Rich et al cited in this manuscript indicates that the highest risk for MASH is in Hispanic populations and lowest in Black populations vs White ones. So, while differences between Black and White populations can exist, these could be much smaller. One may ask how the genes that Dr. Gorlov et al found comparing the two populations relate to the Hispanic one, given that this is the population with the highest MAFLD burden.

There is some variation in estimates of MASLD prevalence in different populations. Meta-analysis suggests roughly 24-29% prevalence in Hispanics in the U.S. (PMID: 28970148) which is higher but still very similar to Non-Hispanic Whites (PMID: 28970148). Estimated prevalence in Blacks is ~12% (PMID: 23512725). Even though the estimated risk depends on method of diagnostic (ultrasound versus serum liver enzymes) and targeted population there is a general agreement that risk is high in U.S. Hispanics and Whites and low in African Americans. We did not include analysis of Hispanics as a separate group because population size was low.

It would be useful to know if the two cohorts analyzed in the manuscript, White and Black individuals, include people living in rural vs urban areas because access to food and its quality can be significantly different.

This is a good point, but unfortunately, we do not have these data.

In Figure 1, please state if the age and BMI are statistically different or not. It seems that there are no statistical differences in these populations, but this needs to be clearly stated.

In the revised manuscript, we provided statistics for age and BMI differences between Blacks and Whites. In both cases, the differences did not reach the level of statistical significance: p=0.09 for age and p=0.25 for BMI.

Are liver biopsies available for some of the cases analyzed? Integrating analyses of liver biopsies with the genes identified as the most different between the two cohorts, may lead to stronger conclusions and a better interpretation of the data.

No, liver biopsies are not available.

In fact, one of the major limitations of this study is the full interpretation of the role of these genes including MMP15, LAMB2, UCN3, PRSS3 among others in White vs Black populations. The interpretation of these data is superficial in the Results and Discussion sections and needs more strengthening. For example, the 9 genes identified in the Results section: Race-specific MASLD-associated genes, shows the list of the genes that result positively or negatively correlated with MASLD, but fails to frame these results in the context of the cases analyzed. As an example, COL5A2 increases correlate with MASLD in White individuals, whereas CXCL9 shows increases in White individuals but no changes in Black individuals and so on... Based on the results of these 9 genes, are White individuals with MASLD more predisposed to progression of disease than Black individuals or not? What do the pathways associated with the genes identified by the authors suggest for the 2 populations?

We substantially extended the Introduction section to justify the need of comparative genetic analysis of MASLD risk and progression for a better understanding of factors contributing to differences between Black and White individuals. We extended the Results section and significantly extended the Discussion section to discuss identified candidate genes contributing

to racial differences for MASLD risk and progression. We added a paragraph on pathway analysis of the identified candidates.

Reviewer 2.

This study reanalyzed publicly available NanoString nCounter gene expression data (GSE163211) from liver biopsies of 98 Black and 211 White individuals undergoing bariatric surgery. The authors have identified race-related differences in hepatic gene expression and how these differences relate to histologically defined stages of metabolic dysfunction-associated steatotic liver disease (MASLD) progression from normal liver, steatosis to MASH without or without fibrosis. Using t-tests, Spearman correlations, two-way ANOVA, FDR control, and pathway/network analyses, the authors report a substantial number of genes differ between racial groups. Consistent with the original study by Subudhi et al., the number of differentially expressed genes increases with more advanced disease stages. Interestingly, the findings indicate that several genes may be race-specific MASLD-associated. The manuscript is well written and provides detailed methodological descriptions; however, several issues related to statistical rigor, threshold justification, interpretation, and limitations require clarification before the conclusions can be fully supported. The authors analyze 800 preselected genes using an FDR < 0.1 threshold. While FDR correction is appropriate for controlling false discoveries, the choice of the 10% cutoff is not explained, which differs from the original study's p < 0.01 threshold by Subudhi et al. This raises concerns about whether the cutoff may influence the number of reported race-specific or progression-associated genes, especially when findings are marginal. A brief justification for selecting FDR < 0.1 is recommended, along with sensitivity analyses (e.g., FDR < 0.05 or adjusted p-values from t-tests/ANOVA) to validate the main conclusions.

We used FDR threshold of 0.1 because our analysis is exploratory and aims to identify and prioritize genes potentially meaningful for racial differences in MASLD risk and progression. Controlling the false discovery rate at 10% allows adequate sensitivity to detect true effects that may be modest in size, which is particularly important when multiple testing reduces statistical power. An FDR of 0.1 is widely accepted in discovery-oriented studies and provides a reasonable trade-off between identifying relevant candidates and limiting the expected proportion of false positives among the reported findings.

- Although the authors acknowledge the substantial imbalance in sample size between Group 4 (13 Black vs. 68 White participants), this disparity likely limits the power and stability of race-by-stage comparisons. The permutation analysis is helpful but does not fully address potential issues such as unstable variance estimates or unreliable interaction testing. A short discussion of how this imbalance affects interpretation, and the inclusion of effect sizes or confidence intervals for key findings, would strengthen the results.

The following paragraph was added: "The smaller number of DE genes observed in Group 4 compared with Group 3 is likely attributable to the markedly unbalanced sample size in Group 4 (68 White vs. 13 Black individuals). Because statistical power is largely driven by the smaller group, this imbalance results in wider confidence intervals and reduced sensitivity to detect true expression differences. In addition, smaller sample sizes are more susceptible to outliers and

random variability, further limiting detection power. Consequently, a direct comparison of p-values across groups with balanced and unbalanced sample sizes may be misleading."

The interpretation of race-specific MASLD-associated genes might be somewhat overstated beyond what the data can support, given the cross-sectional design, lack of covariate adjustment, and absence of functional validation. It would strengthen the manuscript to frame these findings as associative and hypothesis-generating rather than implying causal or mechanistic differences.

The following paragraph was included at the end of the Discussion section: "The findings of this study should be interpreted as associative and hypothesis-generating rather than as evidence of causal or mechanistic differences. Although we observed race-associated variation in hepatic gene expression and enrichment of genes linked to MASLD progression, the observational nature of the analysis precludes direct inference of causality. These results instead provide a framework for future mechanistic studies aimed at elucidating the biological pathways through which race-associated molecular differences may contribute to MASLD susceptibility and progression."

Although the original study discussed several demographic and metabolic covariates, the current re-analysis mainly uses unadjusted t-tests and ANOVA. Without including variables such as age, sex distribution, BMI, and metabolic comorbidities in the statistical models, it is hard to determine whether observed race-associated differences reflect biology or underlying group differences. A brief discussion of this limitation, or inclusion of covariate-adjusted analyses if the metadata permit, would improve interpretability.

Black and White individuals included in this analysis did not differ significantly in age or BMI, and the sex composition was also comparable between the groups. Because individual-level data on clinical comorbidities were not available, we were unable to assess differences in comorbidity burden between groups, which represents a limitation of this study. Accordingly, the following paragraph was added to the revised manuscript: "Although the compared groups did not differ by age, BMI, or sex composition, we were unable to compare them with respect to clinical comorbidities due to the lack of individual-level data. This limitation should be considered when interpreting the results of this study."

The manuscript should clarify whether the 800-gene panel was predetermined, even if this has been reported in the original study, or selected for this reanalysis. Because the analysis focuses on a constrained gene set rather than the full transcriptome, potential selection bias and missed pathways should be briefly acknowledged. A short explanation of how and why this panel was chosen would help readers interpret the scope and limitations of the findings.

The following text was added to the revised manuscript: "Gene expression of 800 genes was analyzed. These genes were selected based on published evidence supporting their roles in liver disease and fibrosis, reflecting a candidate gene approach. To account for the preselected

nature of this gene set and to minimize potential selection bias, we used these 800 genes rather than the entire human genome as the background for gene set enrichment analysis."
