# Supplementary material for: Differences in gene expression may contribute to the racial differences in the risk of MASLD
Source: PLoS One. 2026 May 4;21(5):e0345383. doi: 10.1371/journal.pone.0345383 (PMC13138634; doi:10.1371/journal.pone.0345383)
Supplement: Attachment — Submitted filename: Responses_PLOS ONE_.pdf [file pone.0345383.s006.pdf]

First two reviewers were satisfied with our responses and do not have additional comments and suggestions. In response to the reviewers' 3 comments and suggestions, we conducted additional analyses and have substantially revised the manuscript to improve the clarity and rigor of the study.

Below, we provide a detailed, point-by-point response to reviewer 3 comment and describe how the concern was addressed in the revised manuscript. We have uploaded two versions of the manuscript: a clean version and a version with highlighted changes for ease of review.

Reviewer 1.

Reviewer 1 was satisfied with our responses and did not have any additional comments and suggestions.

Reviewer 2.

Reviewer 2 was satisfied with our responses and did not have any additional comments and suggestions.

Reviewer 3.

1. 800 genes were analyzed based on published evidence. Please cite the relevant reference to support the selection.

We have now included three references to justify the selection of MASLD-associated genes.

2. The language in the manuscript wasn't precise and may be misleading. For example, In the results section, (a) it stated no significant differences for age and bmi while it also stated "slightly higher" and "marginally higher". They conflict with each other; (2) the number of DE genes increased with disease severity, with 41, 141, 316, and 136 ,…, it decreases from group 3 to group 4!

We apologize for the lack of clarity. We agree that phrases such as "slightly higher" or "marginally higher" may be misleading when differences are not statistically significant. We have revised the text to remove such wording and present the results more formally:

"The average age in White individuals was 45.2 ± 1.1 years and in Black individuals 42.4 ± 1.5 years (t = 1.6, p = 0.09). BMI in Black individuals was 48.0 ± 1.1 kg/m² and in White individuals 46.2 ± 0.7 kg/m² (t = 1.2, p = 0.25)."

3. The authors suspected that the smaller number of DE genes in Group 4 compared with Group 3 is due to an unbalanced sample. Can this be checked directly based on the data to

provide further support for this suspicion? Alternatively, the counts can be reported by ancestry/race.

To address this concern, we conducted additional analyses to evaluate the impact of sample size imbalance. Specifically, in Group 3 ("NASH without fibrosis"), we performed permutation analyses by randomly selecting 13 out of 22 Black individuals and comparing them with 49 White individuals. Across 50 permutations, the average number of DE genes was 108.5 ± 4.3, substantially lower than the 316 DE genes identified in the full dataset.

Analysis of mean absolute differences in gene expression between Black and White individuals across consecutive MASLD stages further supports the conclusion that Black versus White divergence in gene expression profiles increases with disease progression. The mean absolute log ratios of gene expression between Black and White individuals were 0.103 ± 0.005 for normal liver histology, 0.121 ± 0.008 for steatotic liver, 0.133 ± 0.005 for NASH without fibrosis, and 0.161 ± 0.007 for NASH with fibrosis.

4. Genes differentially expressed in stage transition were reported. Please clarify the study design on the timeline for gene expression assessment, and stage information and its transition. How to obtain the transition of the stage?

We clarify stage transition analysis in the revised manuscript. The following text was added to the Results section: "Three consecutive transitions associated with MASLD progression were analyzed: (1) the transition from a liver with normal histology to steatotic liver, (2) the transition from steatotic liver to MASH without fibrosis, and (3) the transition from MASH without fibrosis to MASH with fibrosis. We wanted to identify genes differentially expressed between MASLD consecutive stages. We ran the joint analysis with both races together and race-stratified analyses. In the analysis of "all races together", no differentially expressed genes were detected for the first transition."

5. In the joint analysis of race and stage effects. It's unclear about the reported associations. For example, authors reported 232 genes associated with race. As the model contains the interaction, it's clear it refers to the main effect for this association or the joint effect of the main and interaction effect.

We provide the results for main effects (race and stage) and the "race x stage" interaction term. We clarified this in the revision: "We used a two-way ANOVA to estimate the effects of race and stage (categorical variable) on gene expression levels (continuous dependent variable), including a race × stage interaction term in the model. We identified 232 genes for which the main effect of race remained statistically significant in the presence of the race x stage interaction term after adjustment for multiple testing ($p < 0.05$). In contrast, the main effect of stage remained statistically significant for only 56 genes after multiple testing correction. Twenty-six genes were significantly associated with both race and stage, which is comparable to the expected number (16.2) of genes significant for both factors under independence. The results of this analysis are presented in Supplementary Table S4."

6. Preselected genes may limit discovery space and bias the downstream pathway enrichment analysis as well. Are the pathway enrichment analysis results from the preselected 800 genes different from the results from refined gene list?

We acknowledge that restricting the analyses to preselected genes may introduce bias. To reiterate, our choice was limited to these genes, since our paper is a secondary analysis of an existing dataset that included these preselected 800 genes. To mitigate selection bias, we used the same preselected gene set as the background for pathway enrichment analyses. This rationale is emphasized in the revised manuscript.

7. MASLD stage was treated as an ordinal numeric variable for correlation calculation. It might be more suitable to conduct stage-wise contrast instead. Have authors considered conducting ordinal regression?

We thank the reviewer for this important comment. We agree that treating MASLD stage as a continuous numeric variable may not fully capture its ordinal nature. We performed additional analyses to better account for the ordered categorical structure of the outcome.

Following the Reviewer recommendation, we implemented ordinal regression (proportional odds model), which models the ordered nature of MASLD stage. The ordinal regression was run using joint sample (Blacks and Whites together) and separately on Black and Whites individuals. The results of the analyses are shown on 3 additional sheets in supplementary table S3. A total of 76 genes were identified as stage associated in at least one type of analysis. Most of the genes, 73 out of 76, were detected by Spearman's rank correlation. Three genes: CFB, PKLR and UBL5 were detected by ordinal regression but not by correlation analysis. Therefore, the results of ordinal regression analysis are similar and complementary to the results generated by rank correlation and identified genes whose expression level correlates with MASLD progression.

8. It would be informative to formally test the race x stage interactions.

Additional supplementary table S4 was included in the revised paper. The table shows the F statistics and p-values for "race", "stage", and interaction term "race x stage" derived from two-way ANOVA analysis.

9. The sample was based on a bariatric surgery cohort. Please comment on the generalizability of the results.

We have added a "Limitations" section addressing generalizability:

"We acknowledge that our study population was derived from a bariatric surgery cohort, which may limit direct generalizability to the broader population. Individuals undergoing bariatric surgery typically represent patients with more severe obesity and a higher burden of metabolic comorbidities, which may influence disease biology and gene expression patterns (PMID:

41172445, 39920373, 36528638). However, this relatively homogeneous population enables the detection of robust associations. While effect sizes may differ in non-obese populations, we expect the directionality and biological relevance of the findings to extend beyond this cohort. External validation in independent and more diverse populations will be important to confirm generalizability."
